# Non-face-to-face treatment of stress urinary incontinence: predictors of success after 1 year

**DOI:** 10.1007/s00192-016-3050-4

**Published:** 2016-06-03

**Authors:** Anna Lindh, Malin Sjöström, Hans Stenlund, Eva Samuelsson

**Affiliations:** 1 Department of Public Health and Clinical Medicine, Umeå University, Umeå, Sweden; 2 Department of Public Health and Clinical Medicine, Unit for Research, Education and Development—Östersund, Umeå University, Umeå, Sweden

**Keywords:** eHealth, Long-term, Pelvic floor muscle training, Predictors, Self-management, Stress urinary incontinence

## Abstract

**Introduction and hypothesis:**

The objective was to determine predictors of long-term success in women with stress urinary incontinence (SUI) treated with a 3-month pelvic floor muscle training (PFMT) program delivered via the Internet or a brochure.

**Methods:**

We included 169 women with SUI ≥1 time/week who completed the 1-year follow-up (*n* = 169, mean age 50.3, SD 10.1 years). Three outcome variables defined success after 1 year: Patient Global Impression of Improvement (PGI-I), International Consultation on Incontinence Modular Questionnaire Urinary Incontinence Short Form (ICIQ-UI SF), and sufficient treatment. Using logistic regression, we analyzed data from the baseline, and from the 4-month and 1-year follow-ups, for potential predictors of success.

**Results:**

Of the participants, 77 % (129 out of 169) were successful in ≥1 of the outcomes, 23 % (37 out of 160) were successful in all 3. Participants with successful short-term results were more likely to succeed in the corresponding outcome at 1 year than those without successful short-term results (adjusted odds ratios [ORs]: PGI 5.15, 95 % confidence interval [CI] 2.40–11.03), ICIQ-UI SF 6.85 (95 % CI 2.83–16.58), and sufficient treatment 3.78 (95 % CI 1.58–9.08). Increasing age predicted success in PGI-I and sufficient treatment (adjusted OR 1.06, 95 % CI 1.02–1.10, and 1.08, 95 % CI, 1.03–1.13 respectively). Compared with not training regularly, regular PFMT at 1 year predicted success for PGI and sufficient treatment (adjusted OR 2.32, 95 % CI 1.04–5.20, and 2.99, 95 % CI 1.23–7.27 respectively).

**Conclusion:**

The long-term success of a non-face-to-face treatment program for SUI with a focus on PFMT can be predicted by successful short-term results, increasing age, and the performance of regular PFMT after 1 year.

## Introduction

The most common form of incontinence in women is stress urinary incontinence (SUI), affecting 10–35 % of women [1]. Symptoms of SUI are leakage during exertion, coughing, or sneezing [2], and diagnosis before starting non-surgical treatment in an outpatient setting can be based on the symptoms reported by the patient [3, 4]. High BMI, older age, smoking, parity, vaginal delivery, and weakening of the pelvic floor are some of the known risk factors for the development of SUI [5, 6]. The first treatment of choice is pelvic floor muscle training (PFMT) combined with lifestyle interventions, such as weight loss if the person is overweight [7, 8]. The majority of women with SUI report an improvement after 3 months of PFMT [3, 9] and 1 in 3 women becomes continent [4]. The long-term success of PFMT varies between 41 and 85 % [10].

Several studies have investigated the predictors of short-term results after PFMT, but the results have been contradictory. Suggested predictors of a positive result at 3 months are menopause, higher education, no previous urinary incontinence surgery [11], poor contraction strength at baseline, and great improvement in pelvic floor muscle strength [12]. There is limited knowledge on the predictors of long-term outcome, which could be useful for both patients and clinicians for predicting in whom PFMT will be successful and who will need surgery.

In a recent randomized controlled trial (RCT), women with SUI treated with non-face-to face treatment focusing on PFMT were significantly improved at the 4-month follow-up. The improvements were maintained at the 1-year and 2-year follow-ups [13, 14]. The aim of the present study was to evaluate clinically relevant predictors of long-term success after non-face-to-face treatment focusing on PFMT in women with SUI.

## Materials and methods

### Study population

The analyses in this study are based on data from an RCT conducted between 2009 and 2011 (ID: NCT01032265). Women were enrolled in the project via an online screening survey that evaluated the type of incontinence and inclusion and exclusion criteria. The inclusion criteria were: female, age 18–70 years, SUI ≥1 time/week, ability to read and write Swedish, and access to a computer with the Internet. The exclusion criteria were: leakage associated with urgency, previous UI surgery, pregnancy, known malignancy in the lower abdomen, difficulties passing urine, macroscopic hematuria, intermenstrual bleeding, severe psychiatric disorders or Hospital Anxiety and Depression Scale (HADS) score >15 for depression or anxiety, and neurological disease affecting sensitivity in the legs or lower abdomen. Women considered eligible were sent self-assessment questionnaires for additional evaluation, including the validated questionnaires, the Lower Urinary Tract Symptoms Quality of Life (ICIQ-LUTSqol) and the International Consultation on Incontinence Modular Questionnaire Urinary Incontinence Short Form (ICIQ-UI SF), medical history, and a 2-day bladder diary. After their completion, a telephone interview was performed by a urotherapist to confirm the diagnosis of SUI. A total of 250 women aged 18–70 years with SUI ≥1 time/week were randomized by computer-generated block randomization to either an Internet-based training program or a program delivered by post to compare the effect of the two programs after a 3-month treatment period. The programs consisted of PFMT of increasing intensity three times per day for 3 months including contractions for strength and endurance, quick contractions, and the “knack maneuver” with information and lifestyle advice. No face-to-face education was provided before or during the PFMT program. Follow-up was performed at 4 months and 1 year using self-assessed questionnaires. The RCT was described in detail by Sjöström et al. [13].

All women who answered the 1-year follow-up questionnaires were included in the present study (*n* = 169). Participants in both groups achieved highly significant and clinically relevant improvements in the primary outcomes symptom severity and condition-specific quality of life, and the groups did not significantly differ in these measures [13]. In the present study, the analyses include all participants regardless of treatment group. At 1 year, 3 % of the women (5 out of 169, 3 from the postal group and 2 from the Internet group) had undergone surgery for SUI and were also included in the study. None of the women received new medications for incontinence during the study period.

### Definition of success

We used three outcomes to define success after 1 year:

1. Patient Global Impression of Improvement (PGI-I), a validated [15], patient-reported evaluation of the result of treatment consisting of one question: “How is your urinary leakage now compared with before treatment?” On a scale with seven alternatives ranging from very much worse to very much better, participants answering that they were much better or very much better were considered to have a successful outcome.
2. The ICIQ-UI SF is a validated and highly recommended symptom scoring instrument [3, 8, 16] that evaluates symptoms such as frequency, amount of leakage, and overall inconvenience. It consists of four questions: three adding up to a score (0–21 points) and the fourth used to determine the type of incontinence. The minimal important difference (MID) in improvement is the difference between the ICIQ-UI SF score at inclusion and at follow-up. An overall score reduction of 2.5 can be considered clinically relevant [17]. We used ≥3 (rounded up to the closest integer) to define success.
3. “Sufficient treatment” is a question from the follow-up questionnaire: “Do you currently think that the treatment you underwent is sufficient?” answered with “No,” “Yes, I am completely cured from my urinary leakage,” or “Yes, I think the treatment is sufficient even though I am not completely cured.” We considered the two answer alternatives beginning with “yes” as a successful outcome.

### Possible predictors

The possible predictors of long-term success we chose to analyze included known risk factors for SUI [5–7] and suggested predictors from short-term results [11, 12, 18–20]. Because short-term success likely affects long-term success, we included data from the 4-month follow-up [10]. We also included the amount of PFMT performed after 1 year. The data at baseline consisted of age, body mass index (kg/m^2^), education, menopausal status, parity, vaginal delivery of a child ≥4,000 g, local estrogen use, physical activity, time since onset of SUI, previously sought medical contact for SUI, ICIQ-UI SF, condition-specific quality of life as measured by the questionnaire ICIQ-LUTSqol, tea drinking, motivation to perform PFMT (Likert scale 1–10), and self-rated ability to perform PFMT (Likert scale 1–10). The data from the 4-month follow-up consisted of PGI-I, MID ICIQ-UI SF, sufficient treatment, and MID ICIQ-LUTSqol. The data from the 1-year follow-up concerned how often PFMT had been performed in the last 3 months, which could be answered with “Never,” “Sporadically, less than once a week,” “Regularly, 1–3 times/week,” “Regularly, more than 3 times/week,” or “Regularly, daily.”

### Statistical analysis

The baseline variables ICIQ-UI SF, ICIQ-LUTSqol, and age were treated as continuous variables and the other possible predictors as categorical variables. At the 4-month follow-up, the possible predictors PGI-I, ICIQ-UISF, and sufficient treatment were analyzed in the same way as at the 1-year follow-up. The ICIQ-LUTSqol was divided into two categories based on the score reduction between inclusion and the 4-month follow-up; a score reduction exceeding the established MID (i.e., ≥4) [17] was considered a relevant improvement. The categorical variables were divided into categories on the original questionnaire. To have enough participants in each category to proceed with analyses, we had to create fewer categories by adding them together. The categories were reduced based on clinical relevance and the distribution of answers for the following predictors: body mass index (kg/m^2^), education, physical activity, time since onset of SUI, tea drinking, motivation to perform PFMT, self-rated ability to perform PFMT, and how often PFMT had been performed in the last 3 months at the 1-year follow-up. PFMT at 1 year was changed into two categories in which the answers beginning with “regularly” were in one category and “never” or “sporadically, less than one time/week” were another category, as shown in Table 2.

The outcome variables were recoded as 1 for success and 0 for failure. The relationships between each of the potential predictors and the outcomes were tested using the Chi-squared test for categorical variables, independent* t* test for continuous variables, and univariate logistic regression. Age and predictors with* p* < 0.25 were used in a final multivariate model in which non-significant (*p* > 0.05) predictors were manually removed one-by-one starting with the predictor with the highest* p* value, leaving only predictors with* p* < 0.05. The multivariate analysis was also performed without the five women who had undergone surgery.

All statistical analyses were performed using SPSS version 22.0 software.

### Ethics

The study was ethically approved by the Regional Ethical Review Board, Umeå University (number 08-124 M and 2015-79-32 M).

## Results

Of the 169 women included, 1 did not answer the PGI-I, 3 did not complete the ICIQ-UI SF, and 7 did not answer the question about sufficient treatment. The baseline data are summarized in Table 1. The mean age was 50.3 years, less than one-third of patients were overweight or obese, and most women were highly educated. The mean ICIQ-UI SF score was 10.1, which corresponds to moderate severity [21].

**Table 1:** Baseline characteristics of participants who completed 1-year follow-up (*n* = 169)

Characteristic	Data
Mean age, years (SD)	50.3 (±10.1)
BMI, *n* (%)	
< 25	117 (69.2)
25–30	37 (21.9)
> 30	15 (8.9)
Education,* n* (%)	
Primary and secondary	41 (24.3)
Post-secondary	128 (75.7)
Parity, mean (SD)	2.07 (±0.99)
Vaginal delivery of child weighing >4,000 g,* n* (%)	49 (29.0)
Use of local estrogen,* n* (%)	22 (13.0)
Premenopausal,* n* (%)	94 (55.6)
Moderate physical activity,* n* (%)	
< 3 h/week	58 (34.3)
3–5 h/week	54 (32.0)
> 5 h/week	57 (33.7)
Duration of SUI,* n* (%)	
< 1 year	9 (5.3)
1–5 years	70 (41.4)
> 5 years	90 (53.3)
ICIQ-UI SF, mean (SD)	10.14 (±3.20)
ICIQ-LUTSqol, mean (SD)	32.70 (±6.78)
Use of protective pads,* n* (%)	124 (73.4)

*SD* standard deviation, *n* number of observations, *SUI* stress urinary incontinence, *BMI* body mass index (kg/m^2^), *ICIQ*-*UI SF* International Consultation on Incontinence Modular Questionnaire Urinary Incontinence Short Form, *ICIQ-LUTSqol* International Consultation on Incontinence Modular Questionnaire Lower Urinary Tract Symptoms quality of life

### Proportion of successful results after 1 year

**Fig. 1:**
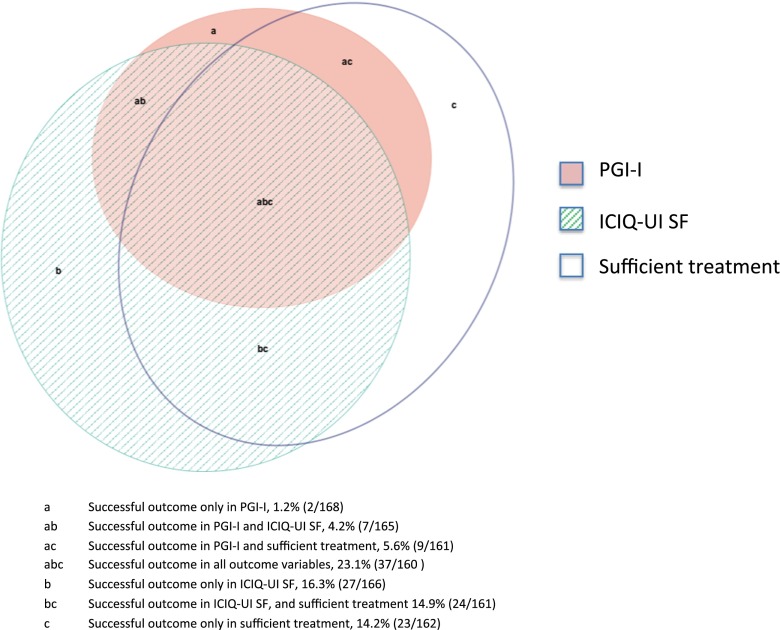
Proportion of patients with successful outcomes in the variables “PGI-I,” “ICIQ-UI SF,” and “sufficient treatment” at 1 year.* PGI-I* Patient Global Impression of Improvement,* ICIQ-UI SF* International Consultation on Incontinence Modular Questionnaire Urinary Incontinence Short Form

Among the study participants, 77 % (129 out of 169) were successful in at least one of the outcome variables. The outcome variables corresponded to the following success rates: PGI-I, 33 % (55 out of 168); ICIQ-UI SF, 57 % (95 out of 166); and sufficient treatment, 57 % (93 out of 162). Twenty-three percent of the women (37 out of 160) were successful in all three outcome variables (Fig. 1).

**Fig. 2:**
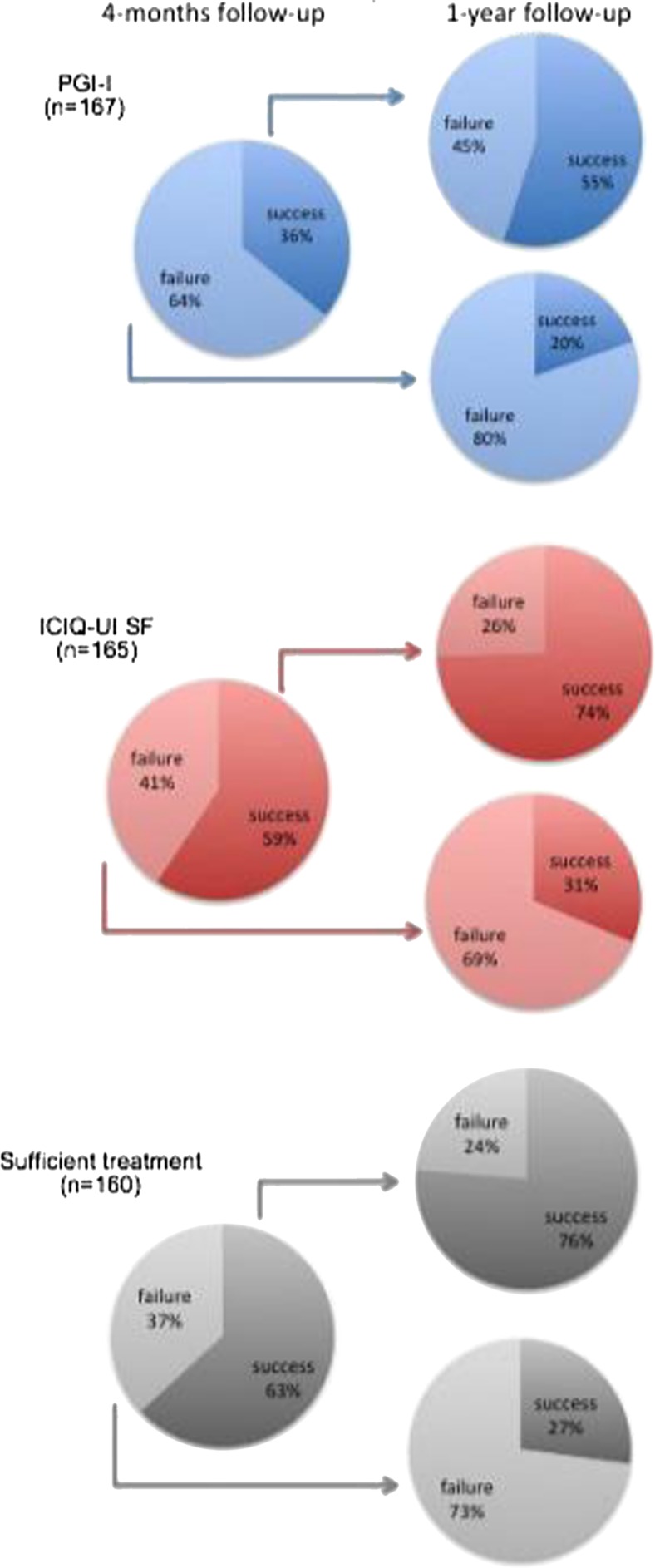
Proportion of participants with success and failure at 4 months and 1 year. The 4-month follow-up only included women who answered at the 1-year follow-up

Of the women who were successful at the 4-month follow-up, 55–76 % were still successful in the corresponding outcome at 1 year. However, 20–31 % of the women who failed at the 4-month follow-up succeeded at the 1-year follow-up (Fig. 2).

### Predictors of a successful outcome after 1 year

The possible predictors time since debut of SUI, previously sought medical contact for SUI, education, and BMI did not result in a p-value <0.25 in any of the outcome variables and were not included in the multivariate analysis. The remainder of the potential predictors had p-values <0.25 in one or more of the outcome variables in the univariate analysis and are presented in Table 2.

**Table 2:** Crude odds ratios (*OR*) for a successful outcome at one year predicted by characteristics at baseline, 4 months, and 1 year

	PGI-I			ICIQ-UI SF			Sufficient treatment		
Predictor	Success (*n* = 55)	Failure (*n* = 113)	OR (95 % CI)	Success (*n* = 95)	Failure (*n* = 71)	OR (95 % CI)	Success (*n* = 93)	Failure (*n* = 69)	OR (95 % CI)
Baseline characteristics									
Age (years)	53.45 ± 9.50*	48.73 ± 10.67*	1.05 (1.02–1.09)*	50.98 ± 10.12**	49.63 ± 10.09**	1.01(0.98–1.05)**	53.68 ± 10.06*	46.04 ± 8.61*	1.09(1.05–1.13)*
Motivation									
1–8	12 (26.1)**	34 (73.9)**	1.00**	15 (34.1)*	29 (65.9)*	1.00***	19 (45.2)**	23 (54.8)**	1.00**
9	9 (28.1)**	23 (71.9)**	1.11 (0.40–3.05)**	21 (65.6)*	11 (34.4)*	3.69 (1.41–9.64)*	18 (58.1)**	13 (41.9)**	1.68 (0.66–4.28)**
10	34 (38.2)***	55 (61.8)***	1.75 (0.80–3.84)***	59 (66.3)*	30 (33.7)*	3.80 (1.77–8.15)*	55 (62.5)**	33 (37.5)**	2.02 (0.96–4.25)**
Self-rated ability to do PFMT									
1–9	25 (28.7)***	62 (71.3)***	1.00***	39 (45.9)*	46 (54.1)*	1.00*	40 (48.8)*	42 (51.2)*	1.00***
10	30 (37.5)***	50 (62.5)***	1.49 (0.78–2.85)***	56 (70.0)*	24 (30.0)*	2.75 (1.45–5.22)*	52 (65.8)*	27 (34.2)*	2.02 (1.07–3.82)*
Vaginal delivery >4,000 g									
No	36 (34.6)**	68 (65.4)**	1.00**	64 (61.5)***	40 (38.5)***	1.00***	53 (52.5)***	48 (47.5)***	1.00***
Yes	13 (26.5)**	36 (73.5)**	0.68 (0.32–1.45)**	22 (46.8)***	25 (53.2)***	0.55 (0.27–1.10)***	29 (63.0)***	17 (37.0)***	1.55 (0.76–3.16)***
Menopause									
No	26 (27.7)***	68 (72.3)***	1.00***	49 (53.8)**	42 (46.2)**	1.00**	39 (43.8)*	50 (56.2)*	1.00*
Yes	27 (39.1)***	42 (60.9)***	1.68 (0.87–3.26)***	42 (60.0)**	28 (40.0)**	1.29 (0.68–2.42)**	51 (75.0)*	17 (25.0)*	3.85 (1.93–7.67)*
Local estrogen									
No	43 (29.9)*	101 (70.1)*	1.00*	79 (56.0)**	62 (44.0)**	1.00**	74 (53.6)*	64 (46.4)*	1.00*
Yes	11 (52.4)*	10 (47.6)*	2.58 (1.02–6.53)*	15 (68.2)**	7 (31.8)**	1.68 (0.65–4.38)**	17 (81.0)*	4 (19.9)*	3.68 (1.18–11.49)*
Moderate physical activity									
< 3 h/week	23 (40.4)***	34 (59.6)***	1.00***	36 (63.2)*	21 (36.8)*	1.00*	33 (57.9)**	24 (42.1)**	1.00**
3–5 h/week	15 (27.8)***	39 (72.2)***	0.57 (0.26–1.26)***	23 (42.6)*	31 (57.4)*	0.43 (0.20–0.93)*	26 (51.0)**	25 (49.0)**	0.76 (0.35–1.62)**
>5 h/week	17 (29.8)***	40 (70.2)***	0.63 (0.29–1.37)***	36 (65.5)**	19 (34.5)**	1.11 (0.51–2.40)**	34 (63.0)**	20 (37.0)**	1.24 (0.58–2.65)**
Tea, cups/day									
None	18 (34.0)**	35 (66.0)**	1.00**	33 (63.5)**	19 (36.5)**	1.00**	29 (56.9)***	22 (43.1)***	1.00***
1–2	24 (28.9)**	59 (71.1)**	0.79 (0.38–1.66)**	44 (53.0)***	39 (47.0)***	0.65 (0.32–1.32)***	43 (51.8)**	40 (48.2)**	0.82 (0.40–1.65)**
≥ 3	13 (40.6)**	19 (59.4)**	1.33 (0.54–3.29)**	18 (58.1)**	13 (41.9)**	0.80 (0.32–1.98)**	21 (75.0)***	7 (25.0)***	2.28 (0.82–6.31)***
ICIQ-UI SF	10.18 ± 3.37**	10.11 ± 3.13**	1.01 (0.91–1.11)**	11.51 ± 2.62*	8.35 ± 2.62*	1.49 (1.30–1.71)*	9.80 ± 3.45***	10.54 ± 2.88***	0.93 (0.84–1.03)***
ICIQ-LUTSqol	33.44 ± 8.40**	32.30 ± 5.85**	1.03 (0.98–1.07)**	34.49 ± 7.18*	30.48 ± 5.55*	1.11 (1.05–1.18)*	32.73 ± 7.63**	32.83 ± 5.79**	0.10 (0.95–1.04)**
4-month follow-up									
PGI-I									
Little better/much worse	21 (19.6)*	86 (80.4)*	1.00*	53 (49.5)*	54 (50.5)*	1.00*	46 (44.2)*	58 (55.8)*	1.00*
Much better/very much better	33 (55.0)*	27 (45.0)*	5.00 (2.49–10.05)*	41 (70.7)*	17 (29.3)*	2.46 (1.24–4.85)*	46 (80.0)*	11 (19.3)*	5.27 (2.46–11.31)*
MID ICIQ-UI SF									
<3	18 (26.5)***	50 (73.5)***	1.00***	21 (31.3)*	46 (68.7)*	1.00*	32 (48.5)***	34 (51.5)***	1.00***
≥3	37 (37.4)***	62 (62.6)***	1.66 (0.84–3.26)***	73 (74.5)*	25 (25.5)*	6.40 (3.22–12.72)*	60 (63.2)***	35 (36.8)***	1.82 (0.96–3.45)***
MID ICIQ-LUTSqol									
< 4	17 (23.3)*	56 (76.7)*	1.00*	30 (41.1)*	43 (58.9)*	1.00*	31 (43.7)*	40 (56.3)*	1.00*
≥ 4	37 (39.4)*	57 (60.6)*	2.14 (1.08–4.23)*	65 (69.9)*	28 (30.1)*	3.33 (1.75–6.33)*	62 (68.1)*	29 (31.9)*	2.76 (1.45–5.25)*
Sufficient treatment									
No	13 (21.7)*	47 (78.3)*	1.00*	30 (50.8)***	29 (49.2)***	1.00***	16 (27.1)*	43 (72.9)*	1.00*
Yes	42 (40.0)*	63 (60.0)*	2.41 (1.16–4.99)*	63 (60.6)***	41 (39.4)***	1.49 (0.78–2.82)***	77 (76.2)*	24 (23.8)*	8.62 (4.14–17.97)*
1-year follow-up									
PFMT last 3 months									
Less than one time/week	30 (26.5)*	83 (73.5)*	1.00*	62 (54.9)**	51 (45.1)**	1.00**	53 (49.1)*	55 (50.9)*	1.00*
Regularly, at least one time/week	24 (45.3)*	29 (54.7)*	2.29 (1.16–4.53)*	33 (63.5)**	19 (36.5)**	1.43 (0.73–2.81)**	40 (75.5)*	13 (24.5)*	3.19 (1.54–6.63)*

Values are presented as means (± standard deviation) or* n* (%)

ORs are the result of univariate logistic regression and are presented with a 95 % confidence interval (*CI*)

*PGI-I* Patient Global Impression of Improvement, *PFMT* pelvic floor muscle training

*p <0.05

**p >0.25

***p <0.25

In the final multivariate regression models (Table 3), a successful result at 4 months for each of the outcome variables predicted a successful result at 1 year for the same outcome variable. Increasing age and performance of regular PFMT during the last 3 months before the 1-year follow-up remained significant predictors in two of the outcome variables. Physical activity had inconsistent results, and there was a trend toward better results with less physical activity in the outcome PGI-I, but the trend was lacking in the outcome ICIQ-UI SF. The severity defined by ICIQ-UI SF at baseline had contradictory results; increased severity at baseline predicted success in the outcome ICIQ-UI SF and less severe leakage at baseline predicted success in the outcome sufficient treatment. The self-rated ability to perform PFMT remained a significant predictor of a successful outcome in one outcome variable.

**Table 3:** Adjusted odds ratios (OR)* for a successful outcome at 1 year

	OR (95 % CI)	*p* value
PGI-I		
Predictive model (Nagelkerke R^2^ 0.30)		
Age (years)	1.06 (1.02–1.10)	<0.001
Moderate physical activity		
< 3 h/week	1.00	
3–5 h/week	0.37 (0.14–0.93)	0.04
> 5 h/week	0.28 (0.11–0.75)	0.01
PGI-I, 4-month follow-up		
Little better/much worse	1.00	
Much better/very much better	5.15 (2.40–11.03)	<0.001
PFMT last 3 months, at 1-year follow-up		
Less than once a week	1.00	
At least once per week	2.32 (1.04–5.20)	0.04
ICIQ-UI SF		
Predictive model (Nagelkerke R^2^ 0.51)		
ICIQ-UI SF, at baseline	1.43 (1.22–1.67)	<0.001
Self-rated ability to perform PFMT		
1–9	1.00	
10	3.04 (1.31–7.08)	0.01
Moderate physical activity		
< 3 h/week	1.00	
3–5 h/week	0.27 (0.10–0.76)	0.01
> 5 h/week	1.02 (0.38–2.76)	0.97
MID ICIQ-UI SF, 4-month follow-up		
< 3	1.00	
≥ 3	6.85 (2.83–16.58)	<0.001
Sufficient treatment		
Predictive model (Nagelkerke R^2^ 0.45)		
Age (years)	1.08 (1.03–1.13)	<0.001
ICIQ-UI SF, at baseline	0.86 (0.75–0.98)	0.03
PGI-I, 4-month follow-up		
Little better/much worse	1.00	
Much better/very much better	3.05 (1.18–7.84)	0.02
Sufficient treatment, 4-month follow-up		
No	1.00	
Yes	3.78 (1.58–9.08)	<0.001
PFMT last 3 months at 1-year follow-up		
Less than 1 time/week	1.00	
Regularly, at least 1 time /week	2.99 (1.23–7.27)	0.02

*Adjusted for age and predictors with a* p* value <0.25 in the univariate logistic regression analysis

The results from the multivariate analysis did not change when the 5 women who had undergone surgery by 1 year were removed from the analysis.

## Discussion

The main predictors of long-term success after non-face-to-face treatment based on PFMT in women with SUI were a successful result at 4 months, the performance of regular PFMT after 1 year, and increasing age. The severity of incontinence at baseline and physical activity had inconsistent results, and the self-rated ability to perform PFMT was only significant in one of the outcome variables.

### Strengths

The population in this study was well defined, as all women had clinically relevant SUI with ≥1 leakage/week and actively sought treatment. PFMT is the first choice for the treatment of women with SUI. The outcome measures ICIQ-UI SF and PGI-I are validated and the ICIQ-UI SF is also highly recommended. The third outcome, sufficient treatment, is important from a clinical perspective and reflects the patient’s goal of treatment, a recommended part of the composite end-points [22]. The possible predictors were chosen based on risk factors or findings from other studies [5, 6, 11, 12, 18–20, 23]. The percentage of women who underwent incontinence surgery during follow-up (3.0 %) was lower in our study than in other long-term studies (4.9–58 %) [10]. We included all women who completed the follow-up, including the 5 who had undergone surgery. We also excluded these 5 women from the final analysis, with no effect on the results.

### Limitations

A possible shortcoming of this study is the lack of an objective outcome measure for the amount or frequency of leakage. We chose to use patient-reported measures based on validated and highly recommended questionnaires. Patient-reported measures are important and correlate with reduced incontinence [24]. The losses to follow-up (32 %) are comparable with the upper range of other long-term studies on incontinence [10]. The women who did not complete the 1-year follow-up were younger and had more severe leakage than the women who completed the follow-up [13]. We do not expect the losses to follow-up to influence our results, because only the women who answered the 1-year follow-up were included in this study. The limited number of participants in this study may increase the risk of type II errors (false-negative), and there may be more predictors that we did not find. The large number of possible predictors that we analyzed could have created significant results by chance.

### Population and treatment

The women in this study were generally highly educated and 75.7 % had a post-secondary education, compared with 35 % of women ≥16 years of age in the general population of Sweden at the time of inclusion (i.e., 2009) [25]. In other fields, studies on online prevention efforts aimed at lifestyle changes have shown that participants are more often white, female, highly educated, and living in high-income countries [26]. This description fits our study population and may be problematic for generalizing the results because of the high education level. In short-term predictor studies, both higher [11] and lower [20] education have been described as predictors of a successful result. However, in our study, education was not a predictor in any of the logistic regression analyses. The NICE guidelines recommend 3 months of supervised PFMT as the first-line treatment for SUI [8]. However, Shamliyan et al. found no significant differences in treatment effects regarding continence rates or discontinuation between supervised PFMT and unsupervised PFMT [3]. The PFMT in this program is unsupervised, but, unlike studies that compare supervised and unsupervised PFMT [27, 28], the women in this study did not receive any face-to-face education by a physiotherapist during the program. Nonetheless, the women in our study population were significantly improved at 4 months, 1 year, and 2 years [13, 14].

### Outcomes and predictors

We found that more severe incontinence was a factor that made women more likely to achieve success based on the ICIQ-UI SF, whereas the women with less severe incontinence were more likely to reach a successful result based on the outcome variable sufficient treatment. The literature on severity as a short-term predictor has reported inconsistent results, with some studies reporting that patients with a higher incontinence frequency have poorer outcomes [11, 18, 20]. However, Hung et al. reported that women with more severe incontinence at baseline are more likely to be successful, based on self-reported results [12]. The choice of outcome variables seems to be very important when analyzing severity as a predictor. The three studies reporting poorer outcomes with more severe incontinence used objective measures, and the study that reported a more successful outcome with increased severity used a subjective outcome measure. When defining treatment outcomes for lower urinary tract symptoms (LUTS), Hilton and Robinson stated that it is important to acknowledge different perspectives. Subjective measures and quality of life were rated highly by the clinician, patient, and any “third party.” Nevertheless, patients also found the objective measures cost reduction and avoidance of surgery important [22].

### Our findings in relation to other findings

Our finding of a successful result at the 4-month follow-up being a predictor of long-term success is comparable with the results in a review on long-term results in which 7 out of 19 studies reported long-term outcome based on short-term success; the responders to the initial program maintained the result better than the non-responders [10]. The results after general resistance training seem to be maintained with one to two training episodes per week after previous involvement in training [29]. We found that regular PFMT at least once per week during the last 3 months at the 1-year follow-up more than doubled the OR of being successful at 1 year for sufficient treatment and PGI-I. Hung et al. found that increased pelvic floor muscle strength, but not PFMT adherence, was a predictor of success [12]. Furthermore, Theofrastous et al. described a reduction in incontinence episodes after PFMT, but the correlation between the improvement and PFMT was weak [23]. In contrast to short-term predictor studies, our results suggest that adherence to PFMT might be important for long-term success.

Our results indicate that increasing age is a predictor of success at the 1-year follow-up in women. No correlation has been found between age and outcome in other predictor studies (short-term) [11, 12, 18–20, 23]. Perhaps in the long-term, our outcomes benefit older patients with regard to more successful results. Younger women may be less accepting of incontinence and more likely to report being unsatisfied with the treatment, which corresponds well with the results of Labrie et al., who found that age ≤55 years is a predictor of surgery after physiotherapy [30]. Another explanation may be that older women have more time to perform the PFMT.

### Clinical implications

More research and longer follow-ups are needed to further evaluate predictors of long-term success and develop specific recommendations for women with SUI. Even though no consensus is available on the optimal training routine, there are recommendations for short-term PFMT [8]. However, studies on training regimens for the maintenance of short-term results in the long term are still missing. Incontinence may affect the physical, psychological, and social well-being of affected individuals [3, 8, 9], but individuals are not equally affected [3]. Therefore, personalized advice would be helpful to provide high-quality treatment. Our results can contribute to predicting who will benefit from the PFMT and what can be done to increase the odds of achieving a successful outcome.

## Conclusion

This study suggests that a successful result after 4 months, the performance of regular PFMT at 1 year, and older age are predictors of long-term success in women with clinically relevant SUI treated with a non-face-to-face training program based on PFMT. Our results can be helpful when informing patients with SUI about treatment based on PFMT in a clinical setting.
